# Dopaminergic modulation of appetitive trace conditioning: the role of D1 receptors in medial prefrontal cortex

**DOI:** 10.1007/s00213-015-3903-4

**Published:** 2015-03-29

**Authors:** M. A. Pezze, H. J. Marshall, H. J. Cassaday

**Affiliations:** School of Psychology, University of Nottingham, University Park, Nottingham, NG7 2RD UK

**Keywords:** Dopamine D1, SKF81297, SCH23390, Medial prefrontal cortex, Prelimbic, Infralimbic, Trace conditioning, Rat

## Abstract

**Rationale:**

Trace conditioning may provide a behavioural model suitable to examine the maintenance of ‘on line’ information and its underlying neural substrates.

**Objectives:**

Experiment la was run to establish trace conditioning in a shortened procedure which would be suitable to test the effects of dopamine (DA) D1 receptor agents administered by microinjection directly into the brain. Experiment lb examined the effects of the DA D1 agonist SKF81297 and the DA D1 antagonist SCH23390 following systemic administration in pre-trained animals. Experiment 2 went on to test the effects of systemically administered SKF81297 on the acquisition of trace conditioning. In experiment 3, SKF81297 was administered directly in prelimbic (PL) and infralimbic (IL) sub-regions of medial prefrontal cortex (mPFC) to compare the role of different mPFC sub-regions.

**Results:**

Whilst treatment with SCH23390 impaired motor responding and/or motivation, SKF81297 had relatively little effect in the pre-trained animals tested in experiment 1b. However, systemic SKF81297 depressed the acquisition function at the 2-s trace interval in experiment 2. Similarly, in experiment 3, SKF81297 (0.1 μg in 1.0 μl) microinjected into either PL or IL mPFC impaired appetitive conditioning at the 2-s trace interval.

**Conclusions:**

Impaired trace conditioning under SKF81297 is likely to be mediated in part (but not exclusively) within the IL and PL mPFC sub-regions. The finding that trace conditioning was impaired rather than enhanced under SKF81297 provides further evidence for the inverse U-function which has been suggested to be characteristic of mPFC DA function.

## Introduction

Working memory has been defined as the capacity to maintain ‘on line’ transitory information in order to allow comprehension, thinking and planning (Goldman-Rakic 1996). Thus, working memory provides a likely mechanism for associative processes in general, and, in particular, when a time interval must be bridged (Gilmartin et al. 2014). With respect to underlying brain substrates, deficits in working memory have been attributed to neuronal loss in medial prefrontal (mPFC) dopamine (DA) pathways (Arnsten et al. 1994; Cai and Arnsten 1997; Goldman-Rakic and Brown 1981; Harada et al. 2002; Robbins and Arnsten 2009). Moreover, there is electrophysiological evidence for the role of mPFC in the temporal integration of experimental stimuli presented in sequences (Fuster et al. 2000). Consistent with the role of DA as a modulator of aspects of mPFC function, DA depletion in the caudate nucleus impaired spatial delayed response task performance (Collins et al. 2000). Conversely, at the appropriate dose, DA D1 receptor agonists can counteract impairments in working memory, as seen for example in aged monkeys (Arnsten et al. 1994; Cai and Arnsten 1997). Whilst as an entity mPFC has been implicated in working memory and temporal processes, the roles of its specific sub-regions in mediating temporal aspects of working memory, and in particular the role of DA within specific sub-regions, have yet to be fully established (Cassaday et al. 2014).

Pavlovian trace conditioning procedures require a subject to learn an association between a conditioned stimulus (CS, e.g. noise) which is separated in time from an unconditioned stimulus (US, e.g. food) outcome (Pavlov 1927). The ability to bridge time delays in a trace conditioning procedure allows animals to nonetheless form associations when potentially causally related events are separated in time. Thus, trace conditioning has been argued to reflect temporal information processing, an aspect of relational learning which represents a key component of working memory (Sweatt 2004). Consistent with the suggestion that trace conditioning procedures tap working memory processes, it is very well established that conventional hippocampal lesions impair trace conditioning, and this effect is reproducible in a variety of conditioning procedures (McEchron et al. 2000; Beylin et al. 2001; Quinn et al. 2002). Moreover, immunohistochemistry for inducible transcription factor has shown dissociable patterns of activation in trace versus delay conditioning in hippocampus (Weitemier and Ryabinin 2004), and *N*-methyl-d-aspartate (NMDA) receptor blockade in the dorsal hippocampus can impair trace conditioning (depending on the trace interval in use; Misane et al. 2005). These studies have all used aversively motivated trace conditioning procedures, but a role for hippocampus in appetitive trace conditioning has also been demonstrated (Chan et al. 2014).

Aspects of working memory, in particular, the maintenance of information on line, are also dependent on the mPFC (Goldman-Rakic 1996; Miller et al. 1996; Levy and Goldman-Rakic 2000; Curtis and D’Esposito 2003). Thus, the role of mPFC in trace conditioning (as well as other aspects of working memory) has also begun to be investigated. Indeed, both lesion (Kronforst-Collins and Disterhoft 1998; Weible et al. 2000; McLaughlin et al. 2002; Han et al. 2003) and electrophysiological (Gilmartin and McEchron 2005; Vidal-Gonzalez et al. 2006) studies have shown a role for mPFC in trace conditioning procedures. Specifically, it has been proposed that the maintenance of attentional resources and working memory allows bridging of the gap between the CS and US during trace conditioning and that this bridging is critically dependent on dorsal mPFC (Gilmartin et al. 2014). A number of studies have compared across mPFC sub-regions including prelimbic (PL) and infralimbic (IL) cortices; for example, evidence for functional differentiation between PL and IL has come from an electrophysiological study showing increased PL and decreased IL neuronal activity during trace conditioning at a 20-s trace interval (Gilmartin and McEchron 2005). Later studies from the same laboratory have provided further evidence consistent with the hypothesis that there are ‘bridging’ cells in PL (Gilmartin et al. 2014). Additionally, there is other evidence to suggest that a role for PL is more readily demonstrated when memory processes are engaged, for example during retention tests (Oswald et al. 2008, 2010; Runyan et al. 2004).

Moreover, there is evidence for dopaminergic modulation of trace conditioning (Nelson et al. 2011; Norman and Cassaday 2003; Cassaday et al. 2005). The present study used trace conditioning as a behavioural model in otherwise normal adult rats in which DA function was manipulated experimentally. Specifically, we examined two different trace intervals in order to test the effects of DA D1 receptor agents at two levels of baseline: (1) using a short (2 s) trace interval consistent with relatively high levels of conditioning which would be suitable to detect impairment and (2) using a longer (10 s) trace interval to produce a lower level of conditioning which would be suitable to detect enhancement (Cassaday et al. 2005, 2008; Kantini et al. 2004). The aims were to identify the role of DA D1 receptors in trace conditioning and to identify any functional differentiation between PL and IL mPFC.

An appetitive trace conditioning procedure was used because appetitive variants are suitable to examine the course of acquisition over a relatively high number of conditioning trials, as well as the distribution of anticipatory responding within the trace interval (Cassaday et al. 2005, 2008; Kantini et al. 2004). Testing conditioning over a number of trials and days in this way requires retention to show improvement (Oswald et al. 2008, 2010; Runyan et al. 2004). This procedure also includes in-built measures to distinguish motor and motivational effects from (changes in) responding reflecting associative learning. This procedure has previously been conducted over 10 or 14 days at ten or eight trials per day (Cassaday et al. 2005, 2008; Kantini et al. 2004) which would be unsuitable for a microinjection study because of the likelihood of mechanical damage around the cannula tips after as many as 10–14 injections. Therefore, experiment 1a was conducted to examine the course of acquisition over a reduced number of training days with the number of trials per day increased to 30 delivered within a longer training session (of just over 60 min). Experiment 1b tested the effects of the DA D1 receptor agonist SKF81297 and the DA D1 receptor antagonist SCH23390 on the expression of learning (in the same rats trained up in experiment 1a). Experiment 1b was also done in order to test for non-specific effects which would be reflected in responding in the inter-trial interval (ITI) and/or responding when the US was delivered and indicative of likely motor and motivational effects, respectively. Experiment 2 examined the effect of systemic SKF81297 on acquisition over 4 days. Experiment 3 went on to test the effects of SKF81297 delivered by microinjection into PL or IL mPFC, on acquisition over 4 days as in experiment 2.

## Materials and methods

### Subjects

On arrival in the laboratory, male Wistar rats (Charles Rivers, UK) were caged in groups of four in individually ventilated cages (IVCs) on a 12:12-h light/dark cycle and given free access to food and water. They were handled daily for 1 week. Experiment 1 used 24 experimentally naive rats (at mean weight 217 g, range 192–240 g) and experiment 2 used 48 rats (at mean weight 378 g, range 337–436 g; drug-naive and counterbalanced for previous behavioural experience). In experiment 3, 48 rats were used: food was freely available until they reached their target pre-operative weight (mean 285 g, range 234–332 g). Rats were weighed daily during the first two post-operative weeks and every 2–3 days thereafter. One rat died during surgery due to complications under anaesthetic; there were three confirmed cases of meningitis post-operatively (these rats were removed from the experiment, and on veterinary advice, Synulox was subsequently administered subcutaneously 0.05 ml/kg to the remaining rats as a prophylactic measure); a further three rats had to be humanely killed when their cannulae became loose; thus, 41 rats completed the behavioural procedures. Food was removed 3 days prior to the start of behavioural procedures, which was 11 days post-operatively in experiment 3, and rats were exposed to sucrose reward pellets in their home cage over 2 days following the introduction of food restriction. Thereafter, rats received 5 g per 100-g bodyweight food ration up to 20 g per day; this ration was adjusted as necessary to allow for healthy weight gain and to stabilise weights once these exceeded 400 g. The home cage ration was additional to the 30 sucrose pellets received during conditioning. Water was available ad libitum. All procedures were carried out in accordance with the principles of laboratory animal care, specifically the UK Animals Scientific Procedures Act 1986, Project Licence number: PPL 40/3716.

### Apparatus

Experimental testing was conducted within a set of four fully automated ventilated conditioning chambers, adapted for appetitive conditioning. The food magazine (recessed in a side wall of each of the chambers) was constantly illuminated whenever food was available. Access to the magazine was via a magazine flap. Nose pokes were recorded by the breaking of the photo beam within the food magazine. The US was one 45-mg sucrose pellet dispensed into the magazine (Formula F, Noyes Precision Food, New Hampshire, UK). Two experimental stimuli were available as potential predictors of food delivery. The target stimulus was a mixed frequency noise (CS), presented via a loudspeaker in the roof of the chamber, set at 72 dB including background and of 5-s duration. An experimental background stimulus was provided by three wall-mounted stimulus lights and the house light flashing on (0.5 s) and off (0.5 s), continuously for the duration of the conditioning session.

### Behavioural procedures

Allocation to conditioning groups (and drug treatments in experiment 1b and experiment 2) was counterbalanced by box. Since experiment 1a confirmed that acquisition was rapid, drug treatments were subsequently limited to 2–4 days. On each day, there were 30 pairings of noise CS and food presented at a 2- or 10-s trace interval.

#### Pre-conditioning

There were 2 days of shaping to accustom rats to eating from the magazine. On the first day, the rats were shaped in pairs; on the second and subsequent days of pre-conditioning, they were placed individually in the conditioning chambers. On each of the first 2 days, rats had access to a pre-load of 15 reward pellets with an additional 15 rewards over 15 min to familiarise rats with the food deliveries. The tray flap door was propped open on days 1 and 2 but was closed on subsequent days, so the rats were then required to nose poke the door open to collect food. Then followed 2 days of baseline sessions, during which there were 30 unsignalled rewards over 60 min, delivered on a variable interval around 2 min, to habituate rats to the sounds produced by food delivery.

#### Conditioning

Depending on the experimental group, the reward (US) was delivered 2 or 10 s after CS offset (in the two different trace groups). Thirty signalled rewards were presented on a variable interval, with the constraint that the ITI was always at least 1.5 times longer than the inter-stimulus interval (ISI) length. Throughout acquisition, the background stimulus (flashing lights) was presented continuously. This continuous presentation also encompassed the 2- or 10-s ISI, as applicable, which added to the overall duration of a 60-min session so that conditioning sessions were of either 61- or 65-min total duration.

Magazine activity reflects Pavlovian conditioning. Thus, the most important dependent variables to assess (effects on) trace conditioning were the number of nose pokes during the 5-s CS and the number of nose pokes during the 2- or 10-s trace interval between CS and US (the ISI). This responding was compared with that seen in the 5 s after the delivery of the US in acquisition and in the remainder of the session (the ITI, which excluded responding in the ISI). In order to examine drug effects on conditioning to the experimental background stimulus in experiments 2 and 3, extinction tests conducted 48 h after conditioning procedures had been completed used 30 5-s presentations of the same flashing light stimulus over 60 min. The number of nose pokes was recorded as above.

### Experiments 1b and 2: systemic injection procedure

Drug doses were based on a previous study run in our laboratory (Nelson et al. 2012). Both SKF81297 and SCH23390 (Tocris, UK) were dissolved in saline (0.9 % NaCl) to provide an injection volume of 1 ml/kg. The final pH was adjusted as necessary, to approximately 7 using 0.1 M NaOH. In experiments 1b and 2, SKF81297 (0.4 or 0.8 mg/kg) or vehicle was injected sub-cutaneously (s.c.) 15 min before conditioning sessions conducted at the 2- or 10-s trace interval (two in the pre-trained rats used in experiment 1b and four sessions in experiment 2). After a 3-day washout period, experiment 1b went on to test the effects of the D1 antagonist SCH23390 (0.025 or 0.05 mg/kg) also administered s.c. 15 min prior to a further two conditioning sessions.

### Experiment 3: implantation of guide cannulae into the mPFC

Rats were anesthetised using isoflurane delivered in oxygen (induction, 4–5 %; maintenance, 1–3 %) and were secured in a stereotaxic frame. The skull was exposed and bregma and lambda were aligned horizontally. Bilateral infusion guide cannulae (the ‘mouse’ model C235GS-5-1.2 of Plastic Ones, Bilaney, UK) consisting of a 5-mm plastic pedestal that held two 26-gauge metal tubes, 1.2 mm apart and projecting 5 mm from the pedestal for the PL and 6 mm for the IL, were implanted through small holes drilled in the skull. The tips of the guide cannulae were aimed 0.5 mm above the injection site in the PL or IL sub-region of prefrontal cortex. The coordinates for PL were 3 mm anterior, ±0.6 mm lateral from bregma and 4.0 mm ventral from the skull surface, and the coordinates for IL were +3 mm anterior, ±0.6 mm lateral from bregma and −5.0 mm ventral from the skull surface (based on pilot surgeries). Cannulae were secured to the skull with dental acrylic and stainless steel screws. Double stylets (33 gauge; Plastic Ones, Bilaney, UK) were inserted into the guides (with no protrusion), and the guides were closed with a dust cap. During the 11-day recovery period, rats were checked daily and habituated to the manual restraint necessary for the drug microinfusions.

### Experiment 3: microinfusion procedure

Rats were gently restrained, and 33-gauge injectors (Plastic Ones, Bilaney, UK) were inserted into the guides. The injector tips extended 0.5 mm below the guides into the PL or IL mPFC, and the injector ends were connected through polyethylene tubing to 5-μl syringes mounted on a microinfusion pump. A volume of 0.5 μl/side of 0.9 % saline or of SKF81297 in saline was then infused bilaterally over 1 min. The movement of an air bubble, which was included in the tubing, was monitored to verify that the injection was successfully infused. The injector remained in place for one additional minute to allow for tissue absorption of the infusion bolus. The injectors were then removed and the stylets replaced. The conditioning session started 10 min after the infusion. The solution of SKF81297 used was dissolved in saline at a concentration of 1 mg/ml. This solution was aliquoted and kept frozen until use (in the present study, not longer than 3 weeks). On the day of infusion, an aliquot was thawed and a part of this solution was diluted to a concentration of 0.05 μg/0.5 μl with saline. Because they were bilateral, microinfusions were administered at a total dose of 0.1 μg in 1.0 μl SKF81297 per rat.

### Design and analysis

Experiment 1a was analysed in simple mixed designs with the between-subjects factor of trace (at two levels, 2 and 10 s) and days (at five levels). Experiments 1b and 2 included the additional between-subjects factor of drug (in each case, at three levels of dose). In experiment 3, there were six experimental groups run in a 2 × 3 factorial design, to examine the effects of trace (at two levels, 2 and 10 s) as a function of microinjection condition (at three levels, vehicle, PL and IL). Initial analyses confirmed no difference between the vehicle injections at PL and IL coordinates so these groups were combined for all subsequent analyses. To assess effects over the course of acquisition, the repeated measures factor was days. Significant three-way interactions were followed up by simple main effects analyses, and further post hocs were by Fisher’s LSD test.

The dependent variable was, in each case, the number of nose pokes into the food magazine. To separate out effects on motor responding or motivation for food reward, similar analyses were conducted on the ITI and US response measures. Additionally, the responding of the animals during the 10-s trace interval between CS offset and US delivery was also examined and tested for any effects of drug condition in experiments 2 and 3. The 10-s ISI was broken down into two second bins of time and analysed using repeated measures ANOVAs by bins and days. In experiments 2 and 3, extinction to the light background was examined using the same design, in this case, in relation to six blocks of five unreinforced stimulus presentations (since there was only 1 day of extinction testing).

## Results

The results reported below detail responding for the different response periods of the trace conditioning task, comparing the effects of SCH23390 and SKF81297 in experiment 1b, examining the effects of systemic SKF81297 on acquisition in experiment 2 and the effects of microinfusions of SKF81297 in PL and IL mPFC in experiment 3.

### Experiment 1a

There was a main effect of days which arose because responding to the CS increased as conditioning progressed, *F*(4,88) = 27.397, *p* < 0.001. More importantly, there was a robust effect of trace, both overall, *F*(1,22) = 11.99, *p* = 0.002, and in interaction with days, *F*(4,88) = 11.405, *p* < 0.001. Follow-up analyses showed significant effects of days in animals conditioned at the 2-s, *F*(4,44) = 24.08, *p* < 0.0001, as well as at the 10-s, *F*(4,44) = 3.90, *p* < 0.01, trace interval. This means that there was evidence for an acquisition function in both trace-conditioned groups and the interaction between trace and days most likely arises because of a difference in the slope of the acquisition function in relation to the trace interval in use. The effect of trace was selective to the measure of associative learning provided by CS responding in that there were no such effects involving trace for responding in the ITI, maximum *F*(1,22) = 1.117, *p* = 0.302, or for responding when food was delivered in the US period, *F*s <1 (Table 1).

**Table 1:** Mean nose poke responding (±SEM) during 5-s presentations of the conditioned stimulus (CS), 5 s after food unconditioned stimulus (US) delivery or in the inter-trial interval (ITI) (*N* = 12/cell)

	CS		US		ITI	
Trace	2 s	10 s	2 s	10 s	2 s	10 s
Day 1	16.33 (±2.53)	15.75 (±3.47)	32.92 (±4.99)	31.25 (±4.40)	192.00 (±30.21)	156.92 (±22.18)
Day 2	36.17 (±4.91)	19.42 (±2.74)	38.08 (±4.85)	36.58 (±4.95)	179.25 (±23.96)	147.92 (±30.50)
Day 3	44.75 (±6.28)	19.25 (±3.21)	37.67 (±5.65)	33.67 (±4.16)	153.50 (±29.90)	160.25 (±40.13)
Day 4	59.67 (±9.88)	25.00 (±3.49)	40.83 (±6.36)	43.67 (±4.38)	192.75 (±46.08)	200.42 (±36.65)
Day 5	76.50 (±11.80)	28.42 (±5.22)	37.08 (±5.97)	41.50 (±5.32)	160.67 (±39.53)	168.67 (±33.06)

### Experiment 1b

#### Effects of SKF81297 in pre-trained animals

ANOVA of CS responding over the 2 days of treatment with SKF81297 showed a main effect of trace, *F*(1,18) = 17.691, *p* = 0.001, because responding was overall higher in the group conditioned at 2 s. There was also an interaction between days and drug, *F*(2,18) = 5.335, *p* = 0.015. Table 2 shows that responding tended to increase on the second day under saline and 0.4 whereas responding tended to drop under 0.8 mg/kg SKF81297. No other effects were significant, maximum *F*(2,18) = 2.585, *p* = 0.103, for the main effect of drug. ANOVA of US responding showed no indication of any non-specific effects under SKF81297, maximum *F*(2,18) = 1.152, *p* = 0.338. However, in the ITI, the main effect of days, *F*(1,18) = 6.333, *p* = 0.022, was accompanied by an interaction between days and drug, *F*(2,18) = 3.966, *p* = 0.037. Table 2 shows that whilst under saline, responding was further increased in the ITI from day 1 to day 2; under SKF81297, responding was lower and further dropped on the second day. The main effect of drug was marginal, *F*(2,18) = 3.505, *p* = 0.052.

**Table 2:** Mean nose poke responding (±SEM) during 5-s presentations of the conditioned stimulus (CS), 5 s after food unconditioned stimulus (US) delivery or in the inter-trial interval (ITI) after treatment with SKF (0.4 or 0.8 mg/kg) (*N* = 4/cell)

Trace	2 s			10 s		
SKF	Saline	0.4 mg/kg	0.8 mg/kg	Saline	0.4 mg/kg	0.8 mg/kg
CS						
Day 1	63.50 (±15.46)	66.750 (±20.70)	43.75 (±11.43)	28.50 (±7.05)	15.75 (±4.50)	16.00 (±4.67)
Day 2	74.50 (±16.04)	76.00 (±18.22)	31.00 (±3.81)	34.00 (±11.02)	16.25 (±6.80)	9.75 (±1.55)
US						
Day 1	29.25 (±11.61)	28.75 (±7.57)	26.00 (±2.80)	30.25 (±0.48)	38.50 (±5.56)	32.25 (±1.80)
Day 2	34.75 (±13.03)	28.50 (±2.33)	21.75 (±1.31)	25.75 (±4.01)	37.00 (±4.02)	29.25 (±2.75)
ITI						
Day 1	323.75 (±144.09)	168.00 (±55.02)	133.50 (±12.33)	182.75 (±36.25)	95.25 (±34.11)	144.00 (±47.84)
Day 2	319.75 (±154.97)	99.75 (±32.80)	48.50 (±11.57)	221.75 (±68.07)	71.50 (±33.93)	77.50 (±16.09)

#### Effects of SCH23390 in pre-trained animals

ANOVA of CS responding over the 2 days of treatment with SCH23390 showed a main effect of days, *F*(1,18) = 8.027, *p* = 0.011, a days by trace interaction, *F*(1,18) = 27.428, *p* < 0.001, a days by drug interaction, *F*(2,18) = 4.666, *p* = 0.023, and a main effect of drug, *F*(2,18) = 15.775, *p* < 0.001. Table 3 shows that in addition to the expected difference in conditioning in the 2- and 10-s trace groups, CS responding was very much reduced under drug. Consistent with motor or motivational impairment, treatment with SCH23390 also reduced responding to collect the food US; this effect was confirmed as a main effect of drug, *F*(2,18) = 18.912, *p* < 0.001, and a drug by days interaction, *F*(2,18) = 4.813, *p* = 0.021, as the effect of drug was even more marked on the second day (Table 3). Consistent with motor impairment, treatment with SCH23390 similarly reduced responding in the ITI, *F*(2,18) = 18.215, *p* < 0.001. There was also an interaction between days and trace on responding in the ITI, *F*(1,18) = 4.670, *p* = 0.044. This arose because generally lower ITI responding in rats conditioned at the 2-s trace interval tended to drop further on the second day of drug testing, whereas generally higher ITI responding in rats conditioned at the 10-s trace interval tended to further increase on the second day. No other effects or interactions were significant, maximum *F*(2,18) = 3.100, *p* = 0.070.

**Table 3:** Mean nose poke responding (±SEM) during 5-s presentations of the conditioned stimulus (CS), 5 s after food unconditioned stimulus (US) delivery or in the inter-trial interval (ITI) after treatment with SCH (0.25 or 0.5 mg/kg) (*N* = 4/cell)

Trace	2 s			10 s		
SCH	Saline	0.025 mg/kg	0.05 mg/kg	Saline	0.025 mg/kg	0.05 mg/kg
CS						
Day 1	67.75 (±24.73)	17.00 (±2.12)	6.50 (±1.94)	35.00 (±5.45)	0.75 (±0.25)	0.25 (±0.25)
Day 2	62.75 (±22.16)	4.75 (±2.59)	0.25 (±0.25)	42.00 (±6.79)	0.50 (±0.29)	0.50 (±0.29)
US						
Day 1	28.00 (±6.87)	21.25 (±5.31)	14.25 (±2.06)	29.00 (±1.83)	23.75 (±2.78)	9.75 (±3.77)
Day 2	27.25 (±4.48)	11.00 (±5.58)	1.75 (±0.85)	26.25 (±2.75)	9.50 (±2.47)	2.75 (±0.85)
ITI						
Day 1	126.25 (±49.72)	36.50 (±13.18)	14.25 (±2.66)	175.75 (±48.32)	21.50 (±5.11)	7.00 (±2.12)
Day 2	100.50 (±26.52)	32.50 (±13.74)	9.00 (±6.14)	209.75 (±50.61)	15.75 (±2.25)	12.75 (±3.40)

### Experiment 2

**Fig. 1:**
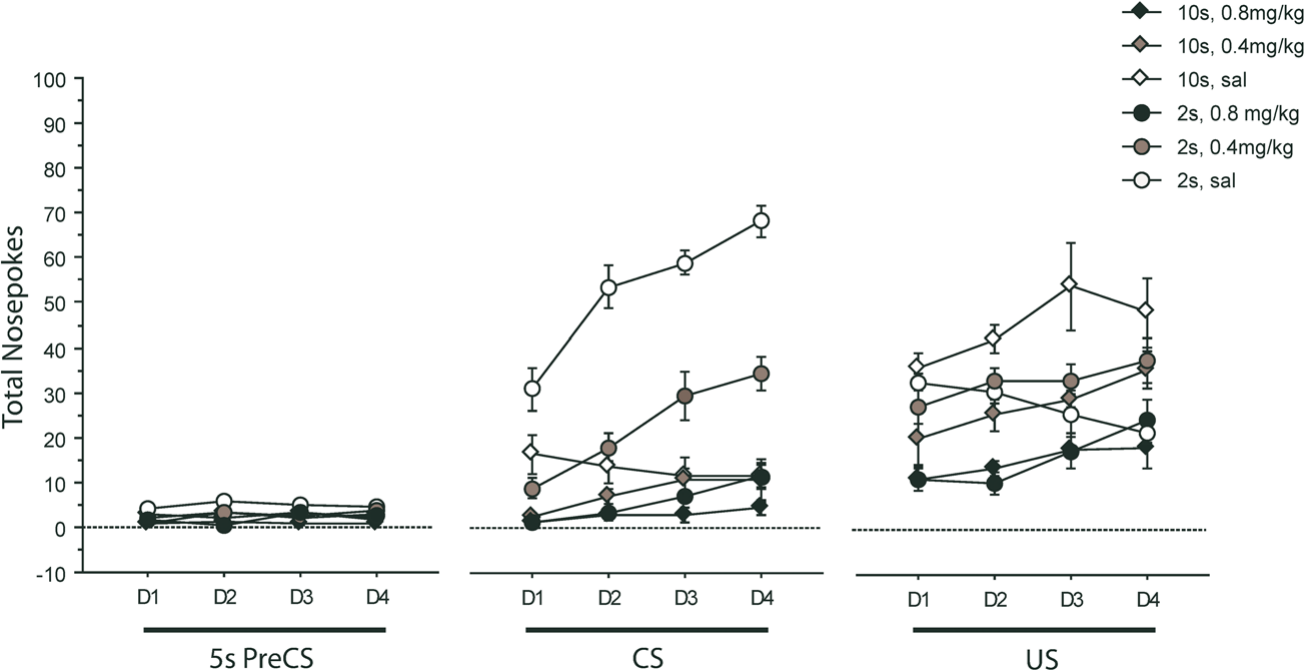
Mean nose pokes are shown as a function of the 4 days of conditioning (*D1* to *D4*) for the 5 s of the inter-trial interval (ITI) just prior to conditioned stimulus (*CS*) presentation (*Pre-CS*) as compared with during the 5-s CS presentation or in the 5-s post-CS when food was delivered (*US*). *Diamonds* denote rats conditioned at the 10-s trace interval, and *circles* denote rats conditioned at the 2-s trace interval after treatment with SKF81297 (s.c.) at 0.8 mg/kg (*black fill*) or 0.4 mg/kg (*grey fill*) in experiment 2. Control rats (*white fill*) were injected with saline

ANOVA of CS responding showed effects of days, *F*(3,126) = 31.908, *p* < 0.001, days by trace, *F*(3,126) = 22.377, *p* < 0.001, days by drug, *F*(6,126) = 2.663, *p* = 0.018, and days by trace by drug, *F*(6,126) = 5.146, *p* < 0.001. There were also significant main effects of trace, *F*(1,42) = 110.986, *p* < 0.001, drug, *F*(2,42) = 85.493, *p* < 0.001, and an overall trace by drug interaction, *F*(2,42) = 35.148, *p* < 0.001. Figure 1 shows that the key three-way interaction arises because the acquisition function seen under saline at the 2-s trace interval was depressed under SKF81297 at both doses. Analysis confined to the 2-s trace group also showed a significant interaction between days and drug, *F*(6,63) = 4.83), *p* < 0.001. Whereas, in the saline group, CS responding increased progressively over the 4 days of testing, this acquisition effect was less pronounced in animals injected with 0.4 mg/kg of the drug and totally absent after injection of 0.8 mg/kg of SKF81297. CS responding was reduced under both doses of SKF81297 and stayed decreased as compared to saline over the four consecutive days of testing (day 1—*F*(2,21) = 25.94, *p* < 0.0001; saline vs 0.4 mg/kg, *p* < 0.0001; saline vs 0.8 mg/kg, *p* < 0.0001; day 4—*F*(2,21) = 69.35, *p* < 0.0001; saline vs 0.4 mg/kg, *p* < 0.0001; saline vs 0.8 mg/kg, *p* < 0.0001). Whilst analysis confined to the 10-s trace group also showed a significant interaction between days and drugs, *F*(6,63) = 2.27, *p* = 0.0478), this effect can be seen to be due to a marginal decrease in the number of nose pokes over days in the saline group (Fig. 1).

Systemic administration of SKF81297 also affected collection of the food US in untrained animals. ANOVA of US responding showed in addition to the effects of days, *F*(3,126) = 8.288, *p* < 0.001, and days by trace, *F*(3,126) = 3.060, *p* = 0.031, interactions of drug by days, *F*(6,126) = 2.197, *p* = 0.047, and drug by days by trace, *F*(6,126) = 3.309, *p* = 0.005. There was also a significant main effect of drug, *F*(2,42) = 19.684, *p* < 0.001, as well as an overall trace by drug interaction, *F*(2,42) = 6.160, *p* = 0.005. Analysis confined to the 2-s trace group showed a significant interaction between days and drug, *F*(6,63) = 5.43, *p* < 0.0001. This interaction can be seen to be due to a reduction in US responding in the saline group over the 4 days of testing which was not seen in drug-treated animals. Follow-up analyses confirmed that the difference in US responding between the saline and 0.8 mg/kg SKF81297 2-s trace-conditioned groups seen on day 1 and day 2 was no longer significant by day 3 of testing (day 1—*F*(2,21) = 13.87, *p* < 0.0001; saline vs 0.8 mg/kg, *p* < 0.0001; day 2—*F*(2,21) = 22.63, *p* < 0.0001; saline vs 0.8 mg/kg, *p* < 0.0001; day 3—*F*(2,21) = 3.27, *p* = 0.06). Analysis confined to the 10-s trace group showed a main effect of drug, *F*(2,21) = 14.64, *p* < 0.0001, but no interaction between drug and days, *F*(6,63) = 1.00, *p* = 0.43. Inspection of Fig. 1 shows that US responding was overall highest in the 10-s saline group and the level of US responding seen under 0.8 mg/kg was low. Thus, saline-treated animals actively seek the food even though they have been poorly conditioned to the tone at the 10-s trace interval, but under 0.8 mg/kg SKF81297, the incentive to nose poke was reduced.

ANOVA of responding in the ITI showed an interaction between days and trace, *F*(3,126) = 3.370, *p* = 0.021, a main effect of trace, *F*(1,42) = 8.463, *p* = 0.006, and, in this case, just a main effect of drug, *F*(2,42) = 12.692, *p* < 0.001. However, this change cannot account for drug effects on CS and US responding which were different by trace interval. Figure 1 shows the 5-s pre-CS portion of the ITI for direct comparison with the responding in the 5 s of CS presentation and 5 s after US delivery.

There was no evidence for any selective effect of drug, on the distribution of responding within the 10-s trace interval, maximum *F*(1,21) = 1.908, *p* = 0.182, for the effect of bins in the linear trend.

Other than an interaction between blocks and drug in the linear trend *F*(2,42) = 3.709, *p* = 0.033, there were no significant effects for responding to the light background stimulus (and response rates were very low).

### Experiment 3

#### Histology

**Fig. 2:**
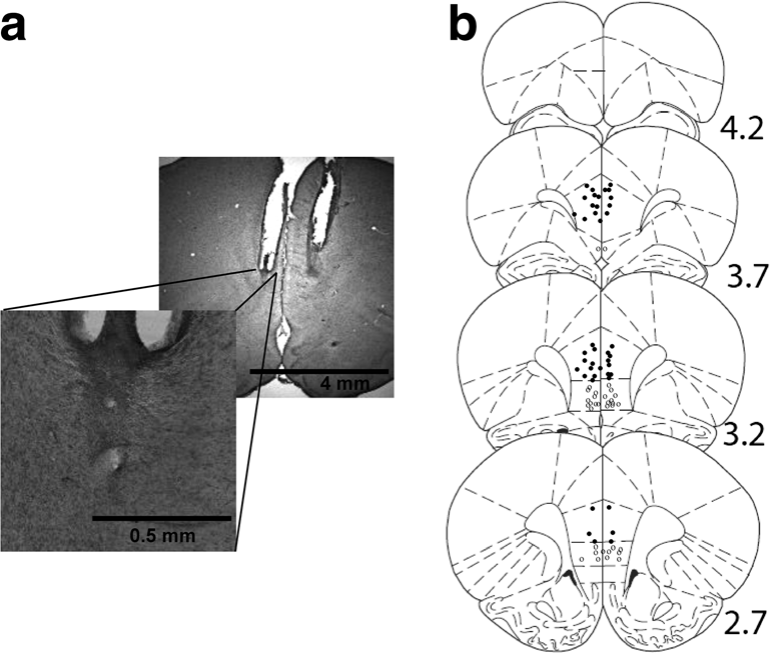
**a** Photograph of a representative placement which illustrates the area around the injection. There is no obvious indication of gliosis as a result of the microinfusions. **b** Approximate locations of infusion cannula tips, in the prelimbic (*black dots*) and infralimbic (*grey dots*) sub-regions of the mPFC. The placements on the borderline with the prelimbic area but which were not clearly infralimbic are also shown in *black*. Placements are shown on coronal plates adapted from Paxinos and Watson (1998), with *numbers* indicating distance from bregma in millimetres

Three rats were excluded on histological grounds. One further rat was excluded because an injection cannula was blocked. This left a final sample size of 37 (*n* = 5–8/cell). Two rats had placements on the borderline of PL and IL, but their exclusion made no difference to the results (see below). As shown in Fig. 2a, there was no evidence of gliosis at the cannula tip. Figure 2b illustrates the full range of cannula placements.

#### Behaviour

Statistical analysis confirmed that within the saline-injected group, the PL or IL site of the injection made no difference to the acquisition function at the different trace intervals, maximum *F*(3,27) = 1.614, *p* = 0.209, for days by trace by injection coordinates. Therefore, animals injected with saline in either PL or IL were combined to form a single control group, compared with SKF81297 injections at these coordinates using the factor of infusion (at three levels: saline; SKF81297 in PL; SKF81297 in IL).

**Fig. 3:**
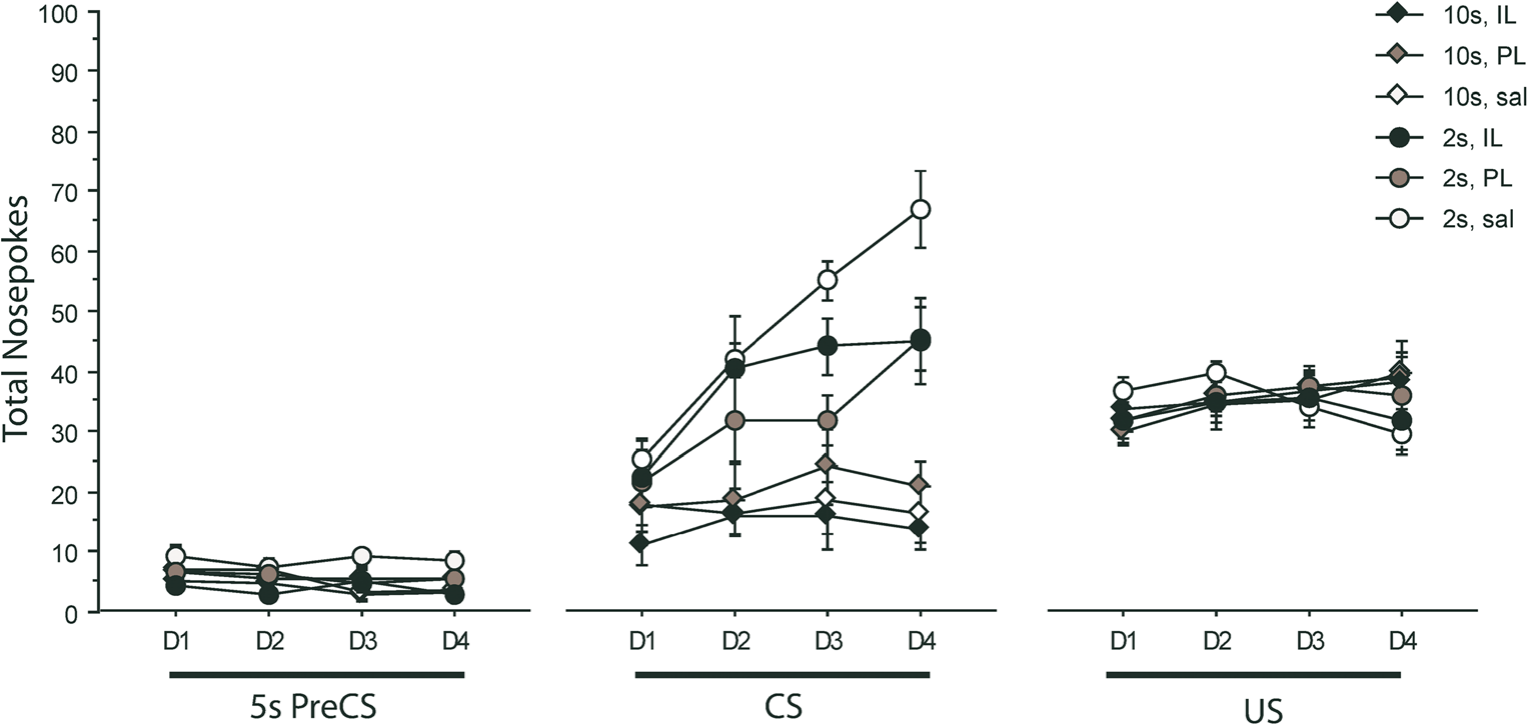
Mean nose pokes are shown as a function of the 4 days of conditioning (*D1* to *D4*) for the 5 s of the inter-trial interval (ITI) just prior to conditioned stimulus (*CS*) presentation (*Pre-CS*) as compared with during the 5-s CS presentation or in the 5-s post-CS when food was delivered (*US*). *Diamonds* denote rats conditioned at the 10-s trace interval, and *circles* denote rats conditioned at the 2-s trace interval after infusion of SKF81297 into infralimbic (*IL*, *black fill*) or prelimbic (*PL*, *grey fill*) regions of medial prefrontal cortex (mPFC) in experiment 3. Control rats (*white fill*) were injected with saline

As would be expected, responding to the CS increased over the 4 days of testing and there was a clear main effect of days, *F*(3,93) = 24.676, *p* < 0.001. The days by trace interaction was also significant, *F*(3,93) = 18.076, *p* < 0.001, because the increase responding was overall greater in the 2-s than in the 10-s trace-conditioned group. The three-way interaction with infusion was significant in the linear trend, *F*(2,31) = 3.399, *p* = 0.046. Figure 3 shows that the acquisition function at the 2-s trace interval was less steep in rats infused with SKF81297 in either the PL or IL sub-region, compared with the saline-infused control group. In line with this lack of difference in relation to mPFC sub-region, excluding the two PL/IL borderline cases made no difference to the interaction, *F*(2,29) = 3.582, *p* = 0.041. Follow-up analysis of the 2-s trace showed that the interaction between days and infusion approached significance, *F*(6,54) = 2.096, *p* = 0.069, while this interaction did not approach significance for the equivalent analysis of the 10-s trace group, *F*(6,39) = 0.53, *p* = 0.77. Together, these analyses suggest that animals infused with SKF81297 into the mPFC reduced learning and the difference by trace identified by the three-way interaction arose because this difference was most apparent in animals conditioned at the 2-s trace interval. Day-by-day analyses of CS responding confirmed that main effects of infusion developed by day 3, *F*(2,18) = 6.431, *p* = 0.007, and day 4, *F*(2,18) = 3.680, *p* = 0.0457. On day 3, the main effect of infusion was due to a difference between saline and PL groups (saline vs PL, *p* = 0.002; saline vs IL, *p* = 0.094), whilst by day 4, both IL- and PL-infused animals nose poked less than controls (saline vs PL, *p* = 0.035; saline vs IL, *p* = 0.026). There was no such effect of infusion on day 1, *F*(2,18) = 0.16, *p* = 0.85, or day 2, *F*(2,18) = 0.61, *p* = 0.55. Thus, infusion effects became apparent over the course of acquisition at the 2-s trace interval. This pattern of results, whereby rats injected with SKF81297 did not reach the same high levels of responding seen in the saline 2-s trace-conditioned group, could be consistent with a motor or motivational effect of SKF81297 infusions which might result in an effective ceiling in responding.

There was a main effect of days on overall ITI responding, *F*(3,93) = 3.734, *p* = 0.014, but there was no indication of any general motor effect of SKF81297 infusions in that there were no significant effects involving infusion on responding in the ITI, maximum *F*(2,31) = 1.095, *p* = 0.347, for the main effect of infusion. Similarly, there was nothing to suggest motivational impairment in that there were no significant effects involving infusion in the US period when food was delivered, maximum *F*(2,31) = 1.045, *p* = 0.364, for infusion by days in the linear trend. We therefore conclude that SKF81297 in PL or IL mPFC attenuated conditioning over a short 2-s trace interval.

Responding distributed within the 10-s trace interval in that there was a main effect of the 2-s time bins, *F*(4,156) = 3.272, *p* = 0.018. However, there was no evidence for any modulation of timing by DA D1 activation in mPFC in that there was no effect of infusion on the distribution of responding within the 10-s trace interval, all *F*s <1. Finally, there were no significant effects in the analyses of responding to the light background stimulus, maximum *F*(1,31) = 2.959, *p* = 0.095, for the main effect of blocks in the linear trend.

## Discussion

Experiment la showed rapid acquisition of associative learning at 30 trials per day. As expected, there was a clear acquisition function for rats conditioned at a 2-s, as compared with at a 10-s trace interval. Experiment lb showed that whilst the administration of SCH23390 resulted in a number of non-specific effects, consistent with impaired motor responding and/or motivation, systemic administration of SKF81297 had relatively little effect in pre-trained animals. Therefore, experiment 2 went on to test the effects of systemically administered SKF81297 on the acquisition of trace conditioning. It was found that systemic SKF81297 depressed acquisition at the 2-s trace interval which was suitable to detect any trace conditioning impairment. The fact that there was no further decrease in conditioning at the 10-s trace interval can be attributed to a floor effect (the 10-s interval was included to test for trace conditioning enhancement). There were a number of non-specific effects, but these did not match the profile of effects on trace conditioning (which depended on the trace interval in use). In experiment 3, SKF81297 (0.1 μg in 1.0 μl) administered directly in PL and IL sub-regions of mPFC similarly impaired appetitive conditioning conducted at the 2-s trace interval. However, this effect was less marked than that seen after systemic administration in experiment 2. There was no evidence within the present study for functional differentiation between PL and IL mPFC in that the same effect was seen, irrespective of injection coordinates.

### Why was trace conditioning impaired rather than enhanced?

There is good evidence that DA D1 activation within mPFC plays an important role in working memory (Arnsten et al. 1994; Cai and Arnsten 1997). The reduction in trace conditioning under SKF81297 observed in the present study stands in apparent contrast to the evidence that DA D1 agonists can restore cognitive function which has been impaired, for example in relation to age or in consequence of neuropathology (Deutch 1993; Dolan et al. 1994; Mizoguchi et al. 2002). Trace conditioning clearly relies on attentional as well working memory processes (Han et al. 2003; Gilmartin et al. 2014). However, SKF81297 administered systemically or by infusion directly into mPFC similarly improves attentional performance measured in the serial reaction time task (Granon et al. 2000). We were unable to demonstrate improved cognitive function in the present study, and this despite the inclusion of a longer trace interval, which should have been suitable to detect cognitive enhancement in that this is most readily demonstrated in cases of low behavioural baseline performance (Granon et al. 2000). Low baseline performance may also occur in consequence of underlying pathology, for example due to ageing (Cai and Arnsten 1997) or due to DA neurodegeneration in Parkinson’s disease (Lange et al. 1992).

This apparent conflict between the results of the present study and previously published findings can be explained in relation to the need to maintain DA within the appropriate physiological range for optimal cognitive function. In particular, there is good evidence to suggest that D1 activity in mPFC follows an inverted U-function. In other words, both hypo- and hyper-activation of these receptors can result in impairments (Arnsten et al. 1994; Arnsten 1998; Cai and Arnsten 1997; Pezze et al. 2014; Zahrt et al. 1997). For example, in normal animals, infusion of SKF81297 into the PL/IL regions of mPFC impaired spatial working memory; this effect was reversible by treatment with SCH23390 and was attributed to supranormal D1 receptor stimulation (Zahrt et al. 1997). Similarly, pretreatment with SCH23390 restored spatial working memory which had been impaired by the anxiogenic FG7142 (Murphy et al. 1996). Thus, whilst DA D1 mPFC-D1 receptor activation clearly modulates working memory, the direction of effects produced by the administration of agonists or antagonists depends on the baseline level of activity and, in particular, whether this is lower or higher than the physiological optimum.

### Were the experimental parameters appropriate?

It is possible that the trace intervals selected for use in the present study were not appropriate to detect the predicted enhancement under SKF81297. In particular, the relatively low levels of learning seen in the rats conditioned at the 10-s trace interval might suggest that this trace interval was too long. However, the point of examining conditioning at the 10-s trace interval was to test for enhanced associative learning under conditions resulting in weak associative learning in untreated controls. Enhanced conditioning over longer trace intervals has been observed after treatment with DA agonists albeit in aversive rather than appetitively motivated procedures of the kind used in the present study (Horsley and Cassaday 2007; Norman and Cassaday 2003). The 2-s interval intended to detect impaired trace conditioning was suitable in that such impairment was clearly demonstrated under SKF81297 in experiments 2 and 3.

The length of the ITI relative to the trace interval has been shown to be an important variable for appetitive trace conditioning and, moreover, a determinant of its sensitivity to hippocampal lesion effects (Chan et al. 2014). In the present study, the ITI was variable and relatively short (at least 1.5 times longer than the ISI length). However, effects of SKF81297 were nonetheless demonstrated.

Another possible limitation arises in that the prolonged conditioning sessions needed to train the animals over fewer days (so that the microinjection study would be viable) may have exceeded the period for which SKF81297 was sufficiently effective. However, this is unlikely to have been a problem in that the effects of D1 agonists have been documented to be maintained for 30 min or more (Mizoguchi et al. 2002; Sorg et al. 2001; Zahrt et al. 1997). Thus, although the half-life of SKF81297 is unclear, based on these previous studies, it is likely to exceed 30 min. Therefore, the treatments used in the present study are likely to retain some effectiveness up to 60 min, albeit this may have been reduced in the latter portion of the session.

### Would the same results be expected in aversively motivated procedures?

Aversive trace conditioning has been more extensively studied, particularly in relation to the role of hippocampus in trace but not delay conditioning (Solomon et al. 1986; Moyer et al. 1990; Gabrieli et al. 1995; Weitemier and Ryabinin 2004; Misane et al. 2005), but with appropriate training parameters, a role for hippocampus in appetitive trace conditioning can also be demonstrated (Chan et al. 2014). Whilst a microdialysis study has shown that ACh release, in both mPFC and hippocampus, was greater during appetitive trace conditioning than during delay conditioning (Flesher et al. 2011), to our knowledge, there has been little previous work on the role of mPFC in appetitive trace conditioning.

Dopaminergic mechanisms are clearly involved in both appetitive (Dalley et al. 2002) and aversive conditioning (Feenstra et al. 2001). However, comparing across appetitive and aversive trace conditioning variants, there is some evidence for differences in the underlying mechanisms. For example, when the effects of electrolytic lesions to nucleus accumbens were compared in appetitive and aversively motivated procedures, effects on trace conditioning were found to depend on whether the procedure in use was aversively or appetitively motivated (Cassaday et al. 2005). DA agonists such as amphetamine and methylphenidate enhanced aversive trace conditioning (Horsley and Cassaday 2007; Norman and Cassaday 2003). However, in an appetitive procedure, the same as that used here apart from being conducted at fewer trials per day over an increased number of days, associative learning was enhanced only in a 0-s (delay) conditioned group and then only when CS responding was corrected for the generally depressed responding seen under these compounds in the ITI (Cassaday et al. 2008). The predicted increase in conditioning to the trace CS was not seen using a similar appetitive trace conditioning procedure (Cassaday et al. 2008; Kantini et al. 2004).

## Conclusions

Experiment 3 identified a role for DA D1 receptors in PL and IL mPFC sub-regions. However, the effects of SKF81297 microinfusion in mPFC were modest compared with those seen after systemic injection. In part, this difference may reflect reduced non-specific effects with more localised administration to mPFC sub-regions. In any case, the profile of motor and motivational changes after treatment with systemic SKF81297 did not match that seen on CS responding. Thus the mismatch between the effects of SKF81297 administered systemically versus directly into mPFC suggests that whilst there is evidence that DA D1 receptors in mPFC modulate trace conditioning, the effects of DA D1 agents are unlikely to be mediated only within the IL and PL mPFC sub-regions examined in the present study. Comparison of the results of experiments 2 and 3 suggests that DA Dl receptors outside of mPFC are also important to trace conditioning.
